# Executive Mechanisms for Thinking about Negative Situations in Both Cooperative and Non-Cooperative Contexts

**DOI:** 10.3389/fnhum.2017.00275

**Published:** 2017-05-24

**Authors:** Azalea Reyes-Aguilar, Juan Fernandez-Ruiz, Erick H. Pasaye, Fernando A. Barrios

**Affiliations:** ^1^Functional Brain Imaging Laboratory, Instituto de Neurobiología, Universidad Nacional Autónoma de MéxicoQuerétaro, Mexico; ^2^Facultad de Medicina, Universidad Nacional Autónoma de MéxicoCiudad de México, Mexico; ^3^Department of Brain and Cognitive Sciences, McGovern Institute for Brain Research, Massachusetts Institute of TechnologyCambridge, MA, United States

**Keywords:** social interaction, emotional valence, mentalizing, fMRI, cooperation

## Abstract

Mentalizing is a fundamental aspect of social cognition that includes understanding the mental states of others. This process involves the participation of a well-defined set of brain regions. However, it is still unknown how different contextual situations, such as previous cooperative or non-cooperative interactions, can modulate the brain activity related to the inference of others’ mental states. Hence, this study investigated whether a previous social interaction can modulate the neural mechanisms involved in a way to response to inferred mental states of cooperators and non-cooperators in positive vs. negative emotional situations. Participants first engaged in a Dictator game with cooperator and non-cooperator confederates. Then, in an fMRI setup, participants had to infer the mental states of the cooperator and non-cooperator confederates under positive and negative situations. Results showed that in addition to the mentalizing network, inferring mental states recruited occipital and cerebellar areas in the cooperative context. A differential pattern of activity that depended on the emotional valence of the situation was also detected, i.e., negative situations recruited prefrontal cortex (PFC) in both contexts, while temporal regions were recruited only for the non-cooperative context. Overall, these results suggest that our previous experiences with others modulate the brain activity related to the inferences we make about their mental states in specific emotional situations.

## Introduction

Human social behavior is largely based on the interpretation of the actions of others in social interaction. This adaptability is built upon cognitive and emotional abilities such as theory of mind or mentalizing (Saxe and Kanwisher, 2003; Saxe and Wexler, 2005; Decety et al., 2012). Mentalizing is the ability to think about the thoughts of other people and create working models of cognitive and emotional states, allowing sharing, predicting and understanding feelings, motivations, and actions (Baron-Cohen, 2002; Blair, 2005; Saxe and Wexler, 2005; Frith and Frith, 2006). This mechanism, which enables successful social interaction, is illustrated by examples of its failure, as in certain instances of autism (Baron-Cohen and Wheelwright, 2004; Frith and Frith, 2006) and schizophrenia (Blair, 2005).

Neuroimaging studies have identified a consistent set of cortical regions associated with mentalizing (Saxe et al., 2009), including the medial prefrontal cortex (M-PFC), precuneous (Prc) and bilateral temporo-parietal junction (TPJ). Medial regions are engaged with self-related cognition (Andrews-Hanna et al., 2010); M-PFC has been associated with self-reference, and Prc with autobiographical memory retrieval in a spontaneous way (Whitfield-Gabrieli et al., 2011), whereas TPJ, mainly in the right hemisphere, is recruited selectively for making inferences about the mental states of others (Saxe and Kanwisher, 2003; Saxe and Powell, 2006) and establishing social contexts for behavior (Carter et al., 2012; Carter and Huettel, 2013).

It has been suggested that the ability to infer the thoughts of other people could be influenced by bottom-up and top-down modulation as an adaptive response to a social contexts (Zaki et al., 2010). Bottom-up processing operates rapidly and involuntarily on sensory inputs of potential importance, and involves neural activity in brain regions sensitive to sensory cues such as visual areas. In contrast, top-down processing which implements longer-term cognitive strategies, is supported by regions more involved in cognition, like the prefrontal cortex (PFC) that participates in the generation of inferences, preparatory set for action, inhibitory control and working memory (Fuster, 2000). Both of these processes are mechanisms fundamental for social cognition (Zaki et al., 2010), for example, during a social interaction a perceiver may have to decide how a social target (e.g., cooperator vs. non-cooperator) feels under a specific emotional situation (e.g., positive vs. negative) based on knowledge from previous interactions with cognitive strategies (top-down processing); such processing also could require integration of relevant sensory information (e.g., visual cues, bottom-up processing) for generating inferences (Rilling and Sanfey, 2011). This information integration occurs in the TPJ and the PFC, the former area acting as a convergence zone for attention, memory, language and social cognition (Carter and Huettel, 2013), while the latter one would bind expectations with dynamic situational context information (McCabe et al., 2001). In addition to visual and prefrontal areas, temporal regions related to representation of semantic script according to the context have been included in the mentalizing network (Frith and Frith, 2003), as have limbic-paralimbic regions (Abu-Akel and Shamay-Tsoory, 2011), and the cerebellum (Stoodley and Schmahmann, 2010; Van Overwalle et al., 2014).

Mentalizing is a cognitive process that support and guides social behavior during interaction with others. These social interactions among humans include cooperation, which has been elevated as an integral part of society (Engemann et al., 2012; Tomasello et al., 2012). Cooperation is understood as an association with others for mutual benefits or goals, which strengthens ties in social interaction with prosocial behavior, e.g., altruism (Tomasello et al., 2012). Cooperation is often associated with feelings of friendship, camaraderie, love, trust, or obligation. In contrast, non-cooperation is associated with rejection and hate that often results in feelings of anger or indignation (Fehr and Schmidt, 2005; Rilling et al., 2008; Engemann et al., 2012). Humans need to distinguish between social contexts (e.g., cooperators vs. non-cooperators) that should be approached or avoided (Frith and Frith, 2012).

A number of cognitive mechanisms are used to deal with this kind of social problem (Frith and Frith, 2012), e.g., executive mechanisms, associated with top-down processing and prefrontal function, resolve conflict by modulating down-stream activity relative to sensory or emotional cues (Fuster, 2000; Zaki et al., 2010). Actually, negative emotional stimuli have been reported to recruit lateral prefrontal regions during effortful top-down control regulatory processes (Mak et al., 2009; Silvers et al., 2015). It has been reported that people who employ more strategies in social cognition tasks have a stronger activation in the superior frontal gyrus (SFG, Rilling and Sanfey, 2011), while the middle frontal gyrus (MFG) has been related to cognitive conflict resolution that involves implementation of fairness norms, and penalizing norm violation (Rilling et al., 2007), and inferior frontal gyrus (IFG) has been related with control of information for identification of threatening stimuli (Heekeren et al., 2006).

Using an fMRI design, in this study we analyzed whether a previous social interaction can modulate the neural mechanisms involved in a way to response to inferred mental states of people who was cooperator or non-cooperator in a previous interaction. Since many real-life situations are emotionally charged and interpreted depending on the contextual appraisal, we evaluated how those modulation mechanisms were involved according to positive vs. negative emotional situations for each context defined by the previous interaction (cooperation vs. non-cooperation). First, contexts were built within the Dictator game outside the scanner in a comfortable play room, where participants were faced with two confederates: one who always used a cooperation strategy, and another who used a non-cooperation strategy. Then, in a following fMRI study, participants were scanned while thinking how cooperative or non-cooperative confederates should feel in situations with negative and positive emotional valence. Because cooperation requires strengthening ties in social interaction in contrast to non-cooperation, which should be just avoided, we hypothesized an increased recruitment of modulation mechanisms for cooperative compared to non-cooperative contexts. Given that negative emotional stimuli recruit top-down control regulatory processes (Mak et al., 2009; Silvers et al., 2015), we also predicted a recruitment of prefrontal areas for negative emotional situations with respect to positive situations, for both contexts.

## Materials and Methods

### Participants

Thirty-four volunteers (17 women) between 25 years and 35 years old (*M* = 28.94, SD = 3.00), all right-handed (Edinburgh Handedness Inventory, Oldfield, 1971, *M* = 42.56, SD = 11.34) and native-Spanish speakers, participated in this study. Participation of women was scheduled during the follicular phase of the menstrual cycle according to their verbal report. No neurological or psychiatric disorders were detected using the Mexican version of the Symptom Check List 90 (Gonzalez-Santos et al., 2007). After the participants were informed of the study procedures and confidentiality they signed the informed consent in accordance with the local ethics committee on the Use of Humans as Experimental Subjects.

### Constructions of Cooperative and Non-Cooperative Contexts

**Figure 1:**
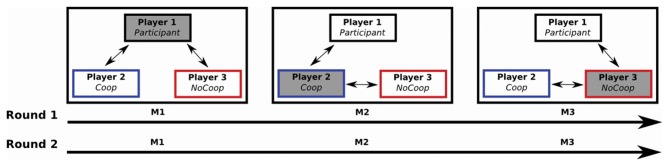
**A sequential iterated Dictator game: one participant and two confederates played two rounds of the game (M1, M2 and M3).** Each M was composed of five iterations. Players received their payments after each iteration. The role of dictator alternated among the three players (filled gray box): in M1, the Participant (player 1) was the dictator (i.e., the player who decided to give or take money from the other two players after their payment); during M2 and M3, players 2 and 3 were the dictators respectively. Players 2 and 3 (confederates) were instructed for perform a specific strategy: player 2 performed a cooperative strategy (Coop), and player 3 a non-cooperative strategy (NoCoop).

First, in a controlled environment close to the institution’s scanners the participants played a sequential iterated Dictator game together with two confederates of the same sex as the participant (Figure 1). Each game consisted of two rounds and in each round the role of dictator was rotated among the three players. Players completed a task (i.e., make a paper case for CDs), and received a payment, the same amount for each player ($4 MXN). After that, the appointed dictator determined the redistribution of the payment of all players, i.e., dictator could give money from her/him own payment to other players (cooperative strategy) or could take money from payment of others two players to herself-himself (non-cooperative strategy). In each game, one confederate was instructed to use a cooperative strategy while the other was instructed to use a non-cooperative strategy for re-distribution of payment.

### Material and Design of Scanning Task

One-hundred and seventy-three emotional situations written in Spanish were selected according to their emotional valence, avoiding those situations of a certain emotional valence that had overlapped in their emotional valence score with other emotional valence (62 positives, 62 negatives and 49 neutrals (Supplementary Material, Table S1) from a previous study (Reyes-Aguilar and Barrios, 2016) to fit a 2 (confederate: Coop vs. NoCoop) × 2 (situations: positive vs. negative) design, resulting in four experimental conditions: the cooperative confederate in positive situations (CPos) and in negative situations (CNeg), and the non-cooperative confederate in positive situations (NCPos) and in negative situations (NCNeg). To later evaluate the participant response to the different confederate situations, a picture was taken of the confederates and of five strangers with the same sex as the participant, all with neutral facial expressions. All pictures were 325 × 480 pixels in size, edited in Adobe Photoshop CS5 to ensure uniformity. Written and informed consent was provided for the use of photographs.

**Figure 2:**
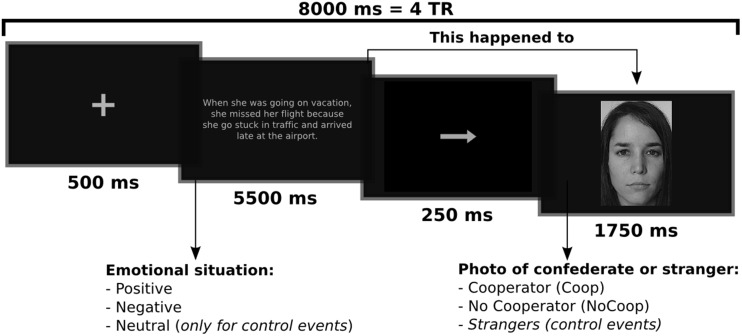
**Example of an experimental trial or event.** Participants read an emotional situation in a text for 5.5 s followed by an arrow for 0.25 s which indicated that this emotional situation happened to people who appeared in the next slide for 1.75 s The interval between trials (fixations) was 0.5 s. Written and informed consent was provided for the use of confederate and non-confederate subjects photographs.

An experimental event trial within the fMRI scanning session consisted of an emotional situation followed by a picture of a confederate or stranger (Figure 2). The emotional situation was presented for 5500 ms, followed by an arrow for 250 ms, and the picture for 1750 ms. Events were separated by 500 ms fixation periods. Events were presented randomly in an event-related paradigm designed using the tool for automatically scheduling events for rapid-presentation event-related (RPER) fMRI experiments^1^. Each run contained 68 events of which five events were for each experimental condition combined with 48 control background events, and lasted 9.5 min. The whole experiment session consisted of four runs. The order of runs was counterbalanced across participants. For each of the four experimental conditions there were a total of 20 events, none of these events were repeated (detailed explanation of events is provided in Supplementary Material Figure S1). Stimuli were presented on a black background via E-Prime 2 (Psychology Software Tools, Inc., Pittsburgh, PA, USA) and MR compatible button system and goggles (NordicNeuroLab, Bergen, Norway) were used as a synchronized projection system.

### Procedure of Scanning Task

One hour after the Dictator game, the scanning process required participants to read emotional situations that happened to confederates or strangers (control background events). Participants were instructed to press a button indicating that they had read the text describing each situation. Then, during the face presentation, they were asked to imagine how that person should feel in the situation that they just read. For the four runs presenting 20 events each. To ensure that the participants read all the text in the allotted time and that they identified the confederates in the photographs, they completed two control tasks in the scanner using the presentation and button response system before the acquisition process: one for reading comprehension and speed and another one for facial identification and recognition in the fMRI experimental context (Supplementary Material, Figures S2–8).

### Post-Scan Questionnaires

After scanning, participants completed a standard Empathy Quotient scale, (EQ, Baron-Cohen and Wheelwright, 2004). Finally, participants indicated the intensity of their liking for the two confederates on a 5-point Likert scale, ranging from 1 (no liking) to 5 (high liking).

### Imaging Acquisition

fMRI imaging was performed on a 3.0T GE MR750 instrument (General Electric, Waukesha, WI, USA) using a 32-channel head coil. Functional imaging included 38 slices, acquired using a T2*-weighted EPI sequence with TR/TE 2000/40 ms, field of view of 25.6 cm, a 64 × 64 matrix and 4-mm slice thickness, resulting in a 4 × 4 × 4 mm^3^ isometric voxel. High-resolution structural 3D-T1-weighted images were acquired for anatomical localization (resolution of 1 × 1 × 1 mm^3^, TR = 2.3 s, TE = 3 ms) covering the whole brain.

### fMRI Data Analysis

MRI data were analyzed using FSL 5 (FMRIB’s Software Library^2^) (Smith et al., 2004). Statistical analysis was performed with FMRI Expert Analysis Tool using FMRIB’s Improved Linear Model (FEAT FILM) Version 5.98. Each participant’s data were motion and slice timing corrected, and normalized onto MNI common brain space (Montreal Neurological Institute, EPI Template, voxel size 2 mm × 2 mm × 2 mm). Data were smoothed using a Gaussian filter (full width half maximum = 6 mm) and high-pass filtered during analysis. Blood oxygen level dependent (BOLD) signal was examined during the facial pictures presentation, when participants were instructed to imagine how that person should feel in emotional situations. Statistical analysis of event-related hemodynamic changes was carried out as per the general linear model (GLM, Friston et al. (1995)). The model included the following regressors: CPos, CNeg, NCPos and NCNeg. GLM also included the emotional situations as regressor of no interest. First-level fMRI analysis data was performed to identify regions that increased BOLD signal intensity for each of the four conditions relative to control events for each run significance threshold criterion of *Z* > 2.3. Since each subject responded to the experimental paradigm four independent runs, to estimate a map of the brain regions involved during the process of inferring thoughts related to each of them, a mid-level analysis was carried out using a fixed-effects model, which averaged the activity of each condition during each of the four runs respect to control events: CPos > control events contrast, CNeg > control events contrast, NCPos > control events contrast, and NCNeg > control events contrast, in all cases control events included stranger in positive, negative and neutral emotional situations (contrasts in which control events were segregated by emotional valence of situation [i.e., positive, negative and neutral] are included in Supplementary Material). With the purpose of test whether a previous social interaction can modulate the neural mechanisms involved in a way to response to inferred mental states of people who was cooperator or non-cooperator in a previous interaction, we conducted a conjunction analyses to compare each context respect to control events: CPos > CNeg > control events contrast, and NCPos > NCNeg > control events contrast, and with a conjunction analyses on the contrast we compared between contexts: CPos > CNeg > NCPos > NCNeg, and NCPos > NCNeg > CPos > CNeg. To test how modulation mechanisms were regulated by positive vs. negative emotional situations for each context, we used the contrasts: CPos > CNeg, and CNeg > CPos for cooperation; and NCPos > NCNeg, and NCNeg > NCPos for non-cooperation. Finally, to identify activations at the group-level we used a third-level analysis using FLAME 1 (FMRIB’s Local Analysis of Mixed Effects) and a cluster with a cluster significance threshold criterion of *Z* > 2.3 with *p* < 0.05 corrected for multiple comparisons with Gaussian Random Field (GRF) for results at the whole-brain level (Worsley et al., 2002).

Region of interest (ROI) analysis was performed on regions that have been consistently implicated in executive-cognitive control (Fuster, 2000; Zaki et al., 2010) i.e., right SFG (R-SFG), left SFG (L-SFG), right MFG (R-MFG), left MFG (L-MFG), right IFG (R-IFG) and left IFG (L-IFG). Based on the whole brain analysis results we also included two ROIs related to the semantic system: right middle temporal gyrus (R-MTG) and left MTG (L-MTG). The ROIs were defined based on previously published criteria (Eldar et al., 2007), which consists of including those voxels within a region with well-defined anatomical boundaries which showed significant activation gains in relation to control levels across all participants (Supplementary Material, Figure S9). Then, for each ROI, average percent signal change was calculated for each condition.

Cortical inflated reconstruction was performed with Freesurfer suite^3^ (Dale et al., 1999). The names of the brain regions reported were derived from the Jülich histological atlas (Eickhoff et al., 2007) and the corresponding coordinates from the MNI152 template.

### Statistical Analysis of Behavioral Measures

Behavioral data were analyzed using R 3.1.2. The distribution of money that participants did during Dictator game was analyzed with a two-way ANOVA repeated measures test (2 confederate × 2 rounds), *post hoc* analyses were Tukey HSD test. To test if there is a difference in participants’ total earnings between the both rounds, we use a paired sample *t* test. Scores of liking scale were compared between confederates with a paired sample *t* test, and a Pearson analysis correlation was used to test if the empathic skills (EQ) and scores of liking scales were related.

## Results

### Analysis of Dictator Game

**Figure 3:**
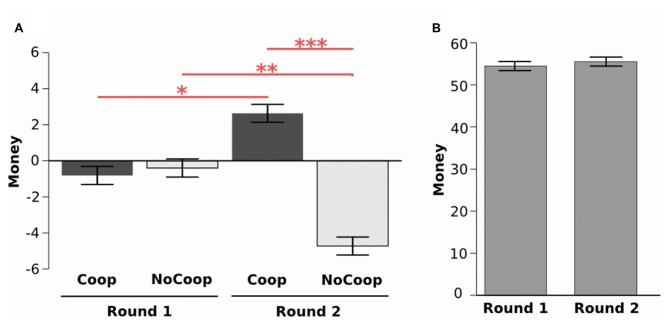
**Results of Dictator game. (A)** Distribution of money during two rounds for each confederate: cooperative (Coop) and non-cooperative (NoCoop). **(B)** Earnings in each of the two rounds. Bars represent the mean, and the error bars show the standard error. *p < 0.05, **p < 0.01, ***p < 0.001.

During the first around participants took money from both confederates (from Coop: *M* = −0.80, SD = 4.16; from NoCoop: *M* = −0.39, SD = 4.26). However, during the second round they gave more money to Coop (*M* = 2.63, SD = 5.48) but removed money from NoCoop (*M* = −4. 72, SD = 6.63). The analysis of these data revealed a significant interaction effect in money distribution (2 confederate × 2 round, *F*_(1,33)_ = 28.69, *p* < 0.001, *η*^2^ = 1.12, ANOVA repeated measures test, Figure 3A). In the second round, participants gave more money to the Coop with respect to the first round (*p* < 0.05, *post hoc* Tukey’s HSD), while removing more money to the NoCoop, in the second round, in relation to the first round (*p* < 0.01). Distribution of money in the second round between confederates was significantly different (*p* < 0.001). Despite the changes in distribution, there were no significant differences in the participants’ total earnings between rounds (round 1: *M* = 54.47, SD = 8.61; round 2: *M* = 55.52, SD = 9.25; *t*_(65.66)_ = 0.488, *p* = 0.627, *d* = 0.11, paired *t*-test, Figure 3B). However, for Coop, total earnings were increased in the second round, and for NoCoop, were decreased (Supplementary Material, Figure S2). Total earnings were significantly different between the three players in each round (Supplementary Material, Figure S10).

### Post-Scan Questionnaires

**Figure 4:**
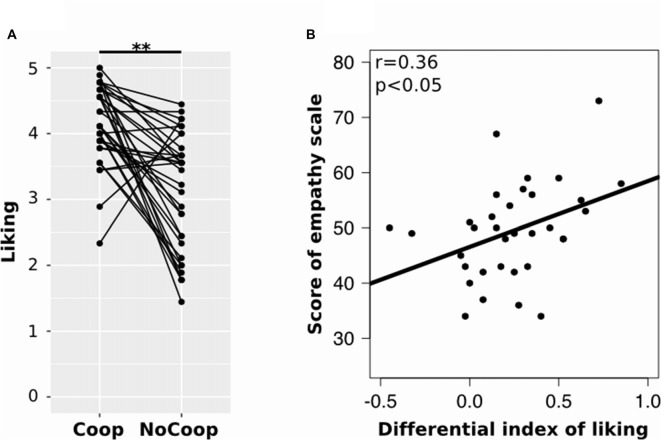
**Results of the post-scan scales. (A)** Post-scan ratings of participants in scale of liking for each confederate: cooperative (Coop) and non-cooperative (NoCoop). Bars represent the mean and the error bars show the standard error. **(B)** Correlation of scores of empathy scale, EQ, with differential index of linking scale. **p < 0.01.

All participants obtained EQ scores within the average range (*M* = 49.41, SD = 8.70). Regarding the confederates’ liking, participants indicated higher liking for Coop (*M* = 4.03, SD = 0.59) than for NoCoop (*M* = 3.07, SD = 0.86; *t* = 3.79, *p* < 0.01, *d* = 1.46 paired *t*-test, Figure 4A). To understand the relationship between liking toward confederates and the empathy scale scores, we calculated a liking differential index (i.e., the difference between liking scores for both confederates) for each participant. This differential liking index was correlated positively with scores in EQ (*r* = 0.36, *p* < 0.05, Pearson test, Figure 4B).

### Whole-Brain BOLD Signal Analysis

An initial whole-brain random effects fMRI analysis identified brain regions that showed significant activation during events involving confederates vs. those involving strangers (control events). Coordinates of the peak activations for all contrasts are shown in Supplementary Material, Table S2.

**Figure 5:**
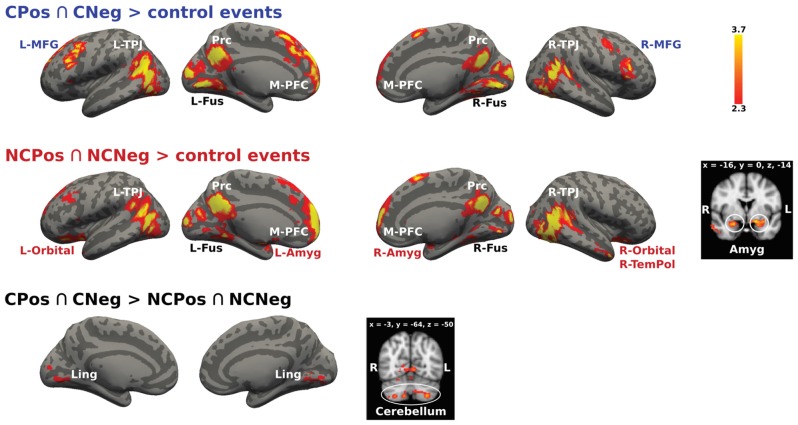
**Activation maps for two confederates.** A whole-brain contrast for cooperative confederate (CPos > CNeg) with respect to control events (CPos > CNeg > control events) revealed increased activity in bilateral TPJ, Prc, medial prefrontal cortex (M-PFC), bilateral cortex fusiform (Fus), and bilateral middle frontal gyrus (MFG). Additionally, contrast for cooperative (NCPos > NCNeg) with respect to control events (NCPos revealed increased activity in bilateral temporoparietal junction NCNeg > control events), includes increased activity in amygdala (Amyg), bilateral orbitofrontal cortex (Orbital), and right temporal pole (R-TemPol). Contrast between confederates, CPos > CNeg > NCPos > NCNeg showed a bigger activity in visual areas and cerebellum for cooperative confederate with respect to the non-cooperative confederate, the inverse contrast (NCPos > NCNeg > CPos > CNeg) did not detect any activation. Color bar indicates *z*-values.

The conjunction analysis designed to identify brain activation related to thinking about mental states of others with whom previous social interaction had been held, (CPos > CNeg > control events and NCPos > NCNeg > control events contrasts), recruited activations in the mentalizing network, i.e., bilateral TPJ, Prc and M-PFC, as well as in the bilateral fusiform gyrus (Figure 5). Further analyses on the effect of the context showed that for the Coop condition there was an activation increase in the PFC (bilateral MFG), while for the NoCoop condition increases were detected in the bilateral amygdala, orbitofrontal cortex and temporal pole (Figure 5).

The analysis of the activation between contexts to test if they resulted in the distinct recruitment of specific modulation mechanisms also showed significant results.

The cooperative context recruited more modulation mechanisms in contrast to the non-cooperative context as follows: CPos > CNeg > NCPos > NCNeg contrast showed increased activity in lingual gyrus and cerebellum, Crus I and II, and lobule VIIa (Figure 5). No significant signal change was detected for the reverse contrast.

**Figure 6:**
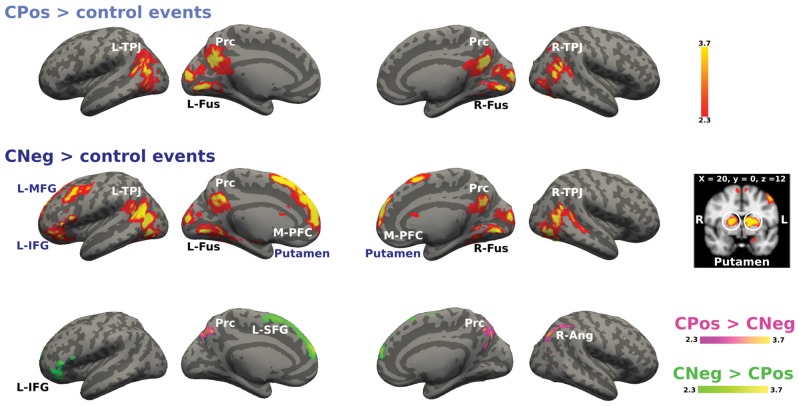
**Activation maps for the cooperative confederate situational events.** A whole-brain contrast for cooperative confederate in positive situations with respect to control events (CPos > control events) revealed increased activity in bilateral TPJ, Prc and bilateral cortex fusiform (Fus). Additionally, contrast for cooperative confederate in negative situations with respect to control events (CNeg > control events), includes increased activity in M-PFC, left inferior and MFG (L-IFG, L-MFG) and putamen. Color bar indicates *z*-values.

We analyzed the activations for positive and negative emotional situations to test if there was a differential recruitment of brain areas related to modulation mechanisms based on the emotional valence of the situation for each context. Figure 6 shows the activation for the cooperative context during positive and negative emotional situations. The main effect CPos > control events contrast revealed higher BOLD response in bilateral TPJ, Prc and bilateral fusiform gyrus. The CNeg > control events contrast showed increased activity in bilateral TPJ, Prc, M-PFC, bilateral fusiform gyrus, bilateral putamen and left prefrontal areas, i.e., MFG and IFG. Finally, the contrast CPos > CNeg resulted in a significant activation change in Prc; while the reverse contrast, CNeg > CPos, resulted in an activation change in L-SFG and L-IFG (Figure 6).

**Figure 7:**
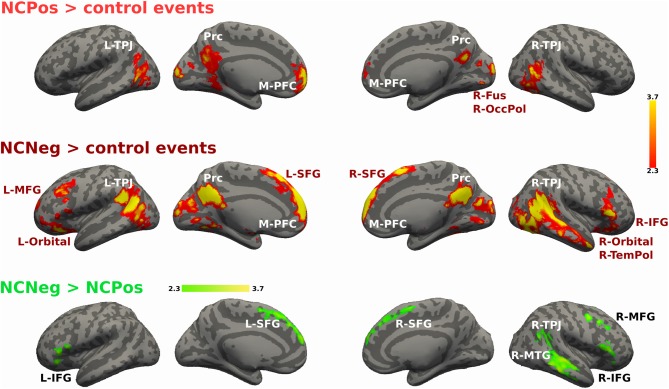
**Activations maps for non-cooperative confederate.** A whole-brain contrast for non-cooperative confederate in positive situations with respect to control events (NCPos > control events) revealed increased activity in bilateral TPJ, Prc, M-PFC and visual areas; right fusiform cortex and occipital pole. Additionally, contrast for non-cooperative confederate in negative situations with respect to control events (NCNeg > control events), includes increased activity in right temporal pole (R-TemPol), bilateral orbitofrontal cortex (Orbital), L-MFG, and bilateral superior frontal gyrus (SFG), and right IFG (R-IFG). Color bar indicates *z*-values.

For the non-cooperative confederate in positive and negative emotional situations the contrast is shown in Figure 7. NCPos > control contrast showed activation increases in bilateral TPJ, Prc, M-PFC, bilateral fusiform gyrus and occipital pole. NCNeg > control events contrast increased activity was detected in bilateral TPJ, Prc, M-PFC, R-TemPol, bilateral orbitofrontal cortex, and left MFG and right IFG. Finally, the contrast NCNeg > NCPos showed signal changes in bilateral SFG and IFG, R-TPJ and the R-MTG (Figure 7). No significant signal change was detected for the reverse contrast.

### ROIs BOLD Signal Analysis

**Figure 8:**
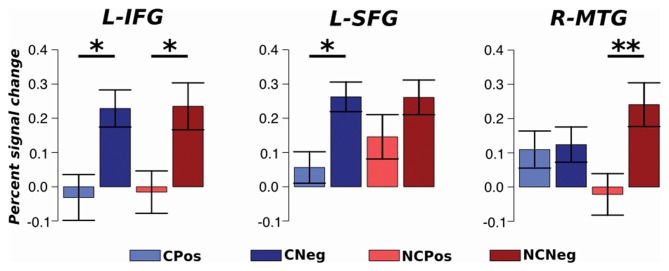
**Region of interest (ROI) analysis.** Average activation with standard error for each ROI; left SFG (L-SFG) and L-IFG. *p < 0.05 and **p < 0.01.

An ROI approach was used to test which brain areas activations were context sensitive between different emotional situations. Average percent signal change values within each ROI were extracted for each condition and analyzed with an ANOVA repeated measures test. The analysis did not detect any region that responded differentially between contexts. However, the analysis detected significant changes depending on the emotional situation. The BOLD activation in L-IFG, which is part of the executive system, was different between emotional situations for both contexts (*F*_(1,33)_ = 13.01, *p* < 0.01, *η*^2^ = 0.14). A *post hoc* analysis, Tukey’s HSD, showed an increased activation in this region during negative respect to positive emotional situations: Coop (*p* < 0.05) and NoCoop (*p* < 0.05, Figure 8). Specifically, for Coop context, activity in L-SFG was higher for negative with respect to positive situations (*F*_(1,33)_ = 28.67, *p* > 0.001, *η*^2^ = 0.27, Figure 8), and a similar pattern was detected in a temporal region related to semantic system, R-MTG, for NoCoop context (*F*_(1,33)_ = 5.91, *p* < 0.05, *η*^2^ = 0.10, Figure 8). No other region showed significant differences between conditions.

## Discussion

Here we tested whether different contextual situations, such as previous cooperation or non-cooperation, modulate the brain activity related to the inferences of others’ mental states in emotionally charged situations. The results showed a common neural activation in the mentalizing network while thinking about the mental states of others for both contexts.

Likewise, thinking about mental states of others in negative situations in both contexts recruited activation in top-down control prefrontal areas associated with dynamic-contextual information (McCabe et al., 2001; Silvers et al., 2015). However, there were also distinct context-dependent brain signal activations; e.g., when thinking about mental states of the cooperator in negative situations, the participants showed increased activity in the SFG associated with top-down control of dynamic contextual information (McCabe et al., 2001; Silvers et al., 2015), while within the non-cooperative context increased activations were detected in the MTG, related to semantic processing (Whitney et al., 2011; Wei et al., 2012).

In this study, the social interaction product of the economic game built a differential context that influenced the behavioral strategy, the subjective response, and the neural activity related to thinking about the mental states of cooperators and non-cooperators. Participants adjusted their strategies during the Dictator game after they knew the confederate’s strategies; without modifying their own payoffs, they were more cooperative with the cooperative confederate and less cooperative with the non-cooperative confederate. Likewise, at the end, participants reported feeling more liking for cooperators than for non-cooperators. Furthermore, greater sensitivity to distinguish subjectively between cooperators and non-cooperators (differential index of liking) was positively correlated with greater empathy abilities (EQ scores).

The fMRI analysis showed activations in the mentalizing network and fusiform face area (FFA) for all experimental conditions, i.e., this temporal region increased its activity whenever participants inferred mental states of people with whom they had had a previous interaction, independently of the emotional valence of the situations (in stranger in positive and negative > stranger in neutral situation contrast was not detected activation in this region), related to face processing (Kanwisher and Yovel, 2006), and association of past experience with them for thinking how they should feel (Frith and Frith, 2012). These results showed that participants were thinking about mental states of other (i.e., confederates) although they did not indicate in what time point the started with this inference.

According to expectations, inferring thoughts of cooperators, with respect to non-cooperators, recruited mechanisms involved of visual-perceptual processing of the occipital fusiform area (OFA), including lingual gyrus, involved in distinguishing between individual faces, e.g., physical aspects of the face (Kanwisher and Yovel, 2006), and mechanisms from cerebellum which has been involved in social cognition, in particular when the level of abstraction is high (Van Overwalle et al., 2014) involving executive functions for planning reciprocity with strategic behavior in cooperation (Stoodley and Schmahmann, 2010). These findings could suggest that cooperative context requires the binding of contingent-perceptual information with a higher level of abstraction that allows evaluating mental states of others to evaluate, promote and strengthen a cooperative relationship (McCabe et al., 2001). The results did not show larger activations for non-cooperative than for cooperative contexts.

Given that many real-life experiences are positive or negative emotionally, this study included emotional situations in both contexts. Positive situations resulted in neural activation in Prc in relation to negative situations only when presented in the cooperative context, in non-cooperative activation was no detected, and in stranger (stranger in positive > stranger in negative situation contrast, Supplementary Material) the increased activity was detected in frontal regions. Activation in Prc has been involved in projecting oneself into the future (Buckner and Carroll, 2007) in an automatic way (Whitfield-Gabrieli et al., 2011). These findings could suggest that for inferring mental states of cooperators in positive situations, automatic mechanisms are recruited for consultation with internally stored information such as autobiographic memory.

Supporting our hypothesis, negative emotional situations recruited brain activity in prefrontal areas, in relation to positive situations, in both contexts, even for strangers (stranger in negative > stranger in positive situations contrast, Supplementary Material). This effect also included the right temporal regions (R-MTG), and R-TPJ for non-cooperative context. These results are supported by previous studies suggesting that PFC is associated with regulatory and evaluative processes (top-down control) of emotional stimuli with negative valence (Koch et al., 2007; Fonville et al., 2014; Lindquist et al., 2016). PFC, as a convergence zone, binds expectations, semantic context information from R-MTG, and social information from R-TPJ (Fuster, 2000; McCabe et al., 2001), that may play a more central role in negative situations respect to positive situations for adaptive behaviors (inhibition, avoiding or prosocial behavior). Positive emotional situation did not show larger activations that negative situations for non-cooperative, which can be related with stimuli presentation, i.e., the emotional situations were presented before the face of confederates, participants could start to think about the emotional valence of situations since it was presented and the response came degraded to face presentation.

The ROI analyses results suggest distinct L-IFG activations depending on the emotional situation, responding with a higher activation for negative in contrast to positive situations. These results confirms previous findings suggesting a prefrontal role in overriding aversive, unpleasant and negative stimulus (Heekeren et al., 2006; Rilling and Sanfey, 2011). Specifically, in the cooperative context this discrimination between emotional situations was detected in L-SFG which has been related to social learning, building a trusting relationship with a strategic processing according to situational context (Rilling and Sanfey, 2011; Silvers et al., 2015). While for non-cooperators, this discrimination between emotional situations was detected in R-TMG, associated with representation of stored conceptual knowledge (Whitney et al., 2011; Wei et al., 2012) as a semantic script according to the context (Frith and Frith, 2003). Inferring mental states in cooperators’ negative situations recruited brain areas related to top-down control, i.e., prefrontal cortex, that could be associated with evaluation of situational-dynamic information for approach or for the investment of future social interactions. For non-cooperators, negative situations seem to require access to stored semantic scripts, i.e., particular settings of activities in a particular context (Frith and Frith, 2003); this may be related to trying to avoid situations previously classified as aversive situations. A more detailed analysis would be necessary to determine how these brain areas interact with the mentalizing network (e.g., psychophysiological interaction). Future research will be necessary to test relationships between social skills, performance in executive functioning and neural activation during social tasks.

This study has some limitations that should be avoided in further studies, as we said above, the emotional situations were presented before the face of confederates, and we analyzed brain response during the face presentation, but we cannot separate the response to emotional situation and face presentations. Although, participants were instructed to think about how that person in the face presentation should feel in that situation, they can think about the situation before of face presentation. Future studies could present emotional situations and person who lived it at the same time and participant can indicate with a behavioral or subjective response in what time point they start to think about mental states of that person in that situation.

Understanding mental states of others could be affected by different factors such as previous social interactions and the emotional content of the specific situation. Together, these results suggest that the mentalizing network is modulated by mechanisms that distinguish between contexts in emotionally charged situations that require integrating information to assign meaning. This distinctive modulation encompasses processes from low level sensory areas to areas involved in higher level top-down control processing.

## Ethics Statement

All our participants signed an informed consent form agreeing to the protocol, which follows the principles expressed in the Declaration of Helsinki and was authorized by the Bioethics Committee of the Neurobiology Institute (Comité de Bioética del Instituto de Neurobiología), UNAM. Those who completed evaluation and interview were recruited for scanning.

## Author Contributions

AR-A and FAB conceived and designed the experiment, AR-A and JF-R contributed to study design, data discussion and wrote the article, AR-A and EHP performed the experiments. FAB revised, contributed and edited the document.

## Conflict of Interest Statement

The authors declare that the research was conducted in the absence of any commercial or financial relationships that could be construed as a potential conflict of interest.

## Footnotes

^1^http://surfer.nmr.mgh.harvard.edu/optseq/

^2^www.fmrib.ox.ac.uk/fsl

^3^http://surfer.nmr.mgh.harvard.edu/
