# Supplementary material for: Executive Mechanisms for Thinking about Negative Situations in Both Cooperative and Non-Cooperative Contexts
Source: Front Hum Neurosci. 2017 May 24;11:275. doi: 10.3389/fnhum.2017.00275 (PMC5442238; doi:10.3389/fnhum.2017.00275)

**Supplementary Material**

**Method**

**Example of one situation with emotional valence**

| **Semantic schema** | **Emotional category** | **Number of words*** |
| --- | --- | --- |
| When she/he was going on vacation, she/he missed her/his flight because she/he got stuck in traffic and arrived late at the airport. | Negative | 18 |
| When she/he was going on vacation, she/he won a free round trip to Cancun on the same airline. | Positive | 16 |
| When she/he was going on vacation, she/he heard the national news on the radio while waiting for her/his flight. | Neutral | 15 |

**Table S1.** Examples of sentences which situations of three emotional categories (positive, negative, and neutral), and the number of words for each situation in Spanish*.

**Detailed explanation of events for scanning task.**

We selected emotional situations from a previous study (Reyes-Aguilar & Barrios, 2016), which had a well-defined emotional valence, without overlapping between different emotional valences. According to this criterion, 173 emotional situations were selected: 62 positives, 62 negatives, and 49 neutrals of which only emotionally charged situations (i.e. positive and negative) were used to experimental condition.

For experimental conditions, from 62 positive situations, 40 were used (i.e. 20 for CPos, and 20 for NCPos), and from 62 negative situations, 40 were used (i.e. 20 for CNeg, and 20 for NCNeg), none of these events were repeated (figure S1).

Therefore, 22 positives, 22 negative and 49 neutrals emotional situations were available as control background events. Each run contained 68 events of which five events were for each experimental condition (i.e. 20 events) combined with 48 control background events. In order to fit 48 control events in each run, a total of 192, some of these situations were repeated once or twice. 20 positive and 20 negative situations were repeated twice; 2 positive and 2 negative situations were repeated once; and 15 neutral situations were repeated once, such that for each kind of emotional situations had 64 as control events (figure S1).


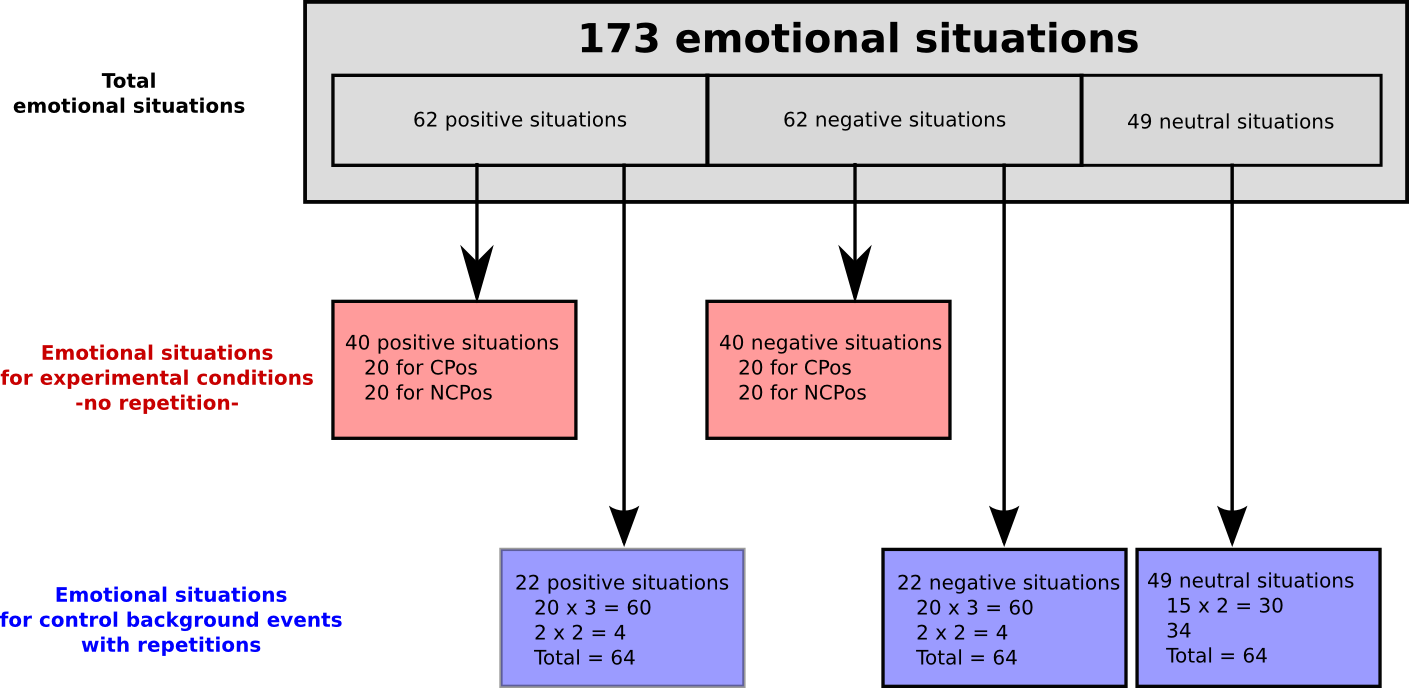


**Figure S1.** Distribution of emotional situations for scanning task.

| **Contrast** | **K** | **Hem.** | **Z-max** | **x** | **y** | **z** | **Anatomical location** |
| --- | --- | --- | --- | --- | --- | --- | --- |
| CPos ∩ CNeg > control events | 17901 | R | 5.58 | 38 | -52 | -22 | Fusiform gyrus |
|  |  |  | 5.32 | 2 | -72 | 2 | Lingual gyrus |
|  |  |  | 4.96 | 50 | -54 | 16 | Angular gyrus |
|  | 6286 | L | 5.07 | -8 | 64 | -4 | Medial prefrontal cortex |
|  |  |  | 4.73 | -6 | 60 | 26 | Superior frontal gyrus |
|  | 1525 | L | 4.42 | -44 | 12 | 44 | Middle frontal gyrus |
|  |  |  | 3.67 | -46 | 2 | 54 | Precentral gyrus |
|  | 1272 | R | 3.97 | 42 | 26 | 22 | Middle frontal gyrus |
|  |  |  | 3.92 | 50 | 28 | 14 | Inferior frontal gyrus, triangular |
|  |  |  | 3.5 | 48 | 0 | 40 | Precentral gyrus |
| NCPos ∩ NCNeg > control events | 9839 | R | 5.37 | 2 | -64 | 24 | Precuneus |
|  |  |  | 4.83 | -54 | -76 | 20 | Left occipital cortex, superior |
|  | 7290 | L | 5.42 | -6 | 60 | 8 | Medial prefrontal cortex |
|  |  |  | 5.39 | -6 | 62 | -2 | Paracingulate gyrus |
|  |  |  | 5.19 | 6 | 58 | 16 | Right superior frontal gyrus |
|  | 5085 | R | 5.36 | 40 | -52 | -22 | Cortex fusiform |
|  |  |  | 5.28 | 48 | -56 | 12 | Middle temporal gyrus |
|  |  |  | 4.25 | 54 | -66 | -2 | Lateral occipital cortex |
|  |  |  | 4.04 | 50 | -40 | 12 | Supramarginal gyrus |
|  | 1667 | L | 4.31 | -16 | -6 | -14 | Amygdala |
|  |  |  | 3.78 | -38 | 26 | -18 | Fusiform gyrus |
|  |  |  | 3.74 | -28 | 8 | -16 | Temporal pole |
|  |  |  | 3.66 | -38 | 28 | -14 | Frontal orbital cortex |
|  | 1283 | R | 4.44 | 52 | 10 | -36 | Temporal pole |
|  |  |  | 3.86 | 30 | 20 | -22 | Frontal orbital cortex |
|  |  |  | 3.77 | 24 | 2 | -20 | Parahippocampal gyrus |
| CPos ∩ CNeg > NCPos ∩ NCNeg | 904 | R | 3.35 | 20 | -58 | -2 | Lingual gyrus |
|  |  |  | 3.2 | 10 | -82 | -10 | Lingual gyrus, posterior |
|  |  |  | 3.04 | 0 | -78 | 4 | Intracalcarine cortex |
|  | 903 | L | 3.29 | -30 | -64 | -50 | Cerebellum, Crus 1 |
|  |  |  | 3.17 | 12 | -74 | -46 | Right VIIb |
|  |  |  | 3.17 | 14 | -78 | -44 | Right Crus II |
|  |  |  | 3.14 | 12 | -66 | -50 | Right VIIIa |

**Table S2. Coordinates of significant activations for all conditions respect to control events (i.e. stranger in positive, negative and neutral situations) and others contrasts.** Peak activations for cooperative (Coop) and non-cooperative (NoCoop) events, for cooperative in positive situations (CPos), cooperative in negative situations (CNeg), non-cooperative in positive situations (NCPos), non-cooperative in negative situations (NCNeg). Hem.: hemisphere, R: right, L: left.

| **Contrast** | **K** | **Hem.** | **Z-max** | **x** | **y** | **z** | **Anatomical location** |
| --- | --- | --- | --- | --- | --- | --- | --- |
| CPos > control events | 6149 | R | 5.48 | 2 | -78 | 0 | Precuneus |
|  |  |  | 5.46 | 2 | -78 | -4 | Lingual gyrus |
|  |  |  | 4.76 | 10 | -98 | 10 | Occipital cortex, posterior |
|  | 3423 | L | 4.54 | -40 | -54 | 14 | Parietotemporal juction |
|  |  |  | 3.95 | -48 | -68 | 16 | Lateral occipital cortex |
|  |  |  | 3.79 | -38 | -56 | -22 | Angular gyrus |
|  |  |  | 3.77 | -46 | -66 | 30 | Lateral occipital cortex |
|  | 3358 | R | 4.58 | 38 | -46 | -26 | Fusiform gyrus |
|  |  |  | 3.86 | 38 | -60 | 34 | Lateral occipital cortex |
| CNeg > control events | 19305 | R | 5.75 | 38 | -52 | -24 | Fusiform gyrus |
|  |  |  | 5.64 | 8 | -58 | -44 | Cerebellum, right IX |
|  |  |  | 5.22 | 30 | -80 | -36 | Cerebellum, right Crus I |
|  |  |  | 4.99 | 2 | -88 | 10 | Supracalcarine cortex |
|  | 7183 | L | 6.89 | -8 | 60 | 26 | Medial prefrontal cortex |
|  |  |  | 5.83 | -8 | 52 | 36 | Superior frontal gyrus |
|  | 3979 | L | 4.47 | -58 | 20 | 10 | Inferior frontal gyrus |
|  |  |  | 4.44 | -38 | 22 | -30 | Frontal orbital cortex |
|  |  |  | 4.29 | -20 | 4 | 10 | Left putamen |
|  |  |  | 4.13 | -8 | 14 | 10 | Left caudate |
|  | 1404 | L | 4.40 | -46 | 16 | 48 | Middle frontal gyrus |
|  |  |  | 3.8 | -50 | 0 | 50 | Left precentral gyrus |
|  |  |  | 2.66 | -40 | 20 | 20 | Inferior frontal gyrus |
|  | 979 | R | 4.15 | 20 | -4 | 12 | Putamen |
|  |  |  | 2.89 | 6 | 20 | 16 | Cingulate gyrus |
|  |  |  | 2.84 | 8 | -6 | 0 | Right thalamus |
|  |  |  | 2.66 | 10 | 10 | 2 | Right caudate |
| CPos > CNeg | 2030 | R | 3.54 | 12 | -66 | 44 | Precuneus |
|  |  |  | 3.61 | 36 | -60 | 34 | Right lateral occipital cortex |
|  |  |  | 3.17 | 38 | -54 | 42 | Left angular gyrus |
| CNeg > CPos | 2594 | L | 5.22 | -10 | 58 | 24 | Superior frontal gyrus |
|  |  |  | 3.2 | -4 | 2 | 56 | Supplementary motor cortex |
|  | 1151 | L | 4.10 | -56 | 22 | 2 | Inferior frontal gyrus |
|  |  |  | 3.69 | -44 | 32 | -12 | Frontal orbital cortex |
|  |  |  | 3.44 | -44 | 24 | -18 | Frontal orbital cortex |

**Table S2 [continuation]. Coordinates of significant activations for all conditions respect to control events (i.e. stranger in positive, negative and neutral situations) and others contrasts.** Peak activations for cooperative (Coop) and non-cooperative (NoCoop) events, for cooperative in positive situations (CPos), cooperative in negative situations (CNeg), non-cooperative in positive situations (NCPos), non-cooperative in negative situations (NCNeg). Hem.: hemisphere, R: right, L: left.

| **Contrast** | **K** | **Hem.** | **Z-max** | **x** | **y** | **z** | **Anatomical location** |
| --- | --- | --- | --- | --- | --- | --- | --- |
| NCPos > control events | 2117 | R | 4.71 | 42 | -50 | -22 | Fusiform gyrus |
|  |  |  | 4.16 | 46 | -66 | -10 | Lateral occipital cortex |
|  |  |  | 3.9 | 58 | -60 | 6 | Middle temporal gyrus |
|  | 1980 | R | 3.92 | 2 | -62 | 22 | Precuneus |
|  |  |  | 3.67 | -8 | -48 | 2 | Cingulate gyrus, posterior |
|  | 1937 | L | 4.68 | -8 | 62 | 4 | Medial prefrontal cortex |
|  |  |  | 2.68 | -10 | 44 | 8 | Cingulate gyrus, anterior |
|  | 1363 | L | 3.98 | -48 | -64 | 4 | Parietotemporal juction |
|  |  |  | 3.84 | -54 | -76 | 20 | Lateral occipital cortex |
|  | 1047 | R | 4.63 | 8 | -98 | 8 | Occipital pole |
| NCNeg > control events | 13183 | R | 5.84 | 2 | -64 | 26 | Precuneus |
|  |  |  | 5.78 | 48 | -54 | 18 | Right angular gyrus |
|  |  |  | 5.5 | 50 | -44 | 14 | Right supramarginal gyrus |
|  | 7219 | R | 6.10 | 0 | 56 | 30 | Medial prefrontal cortex |
|  |  |  | 4.78 | -10 | 32 | 50 | Left Superior frontal gyrus |
|  | 4680 | L | 5.11 | -60 | -56 | 22 | Parietotemporal juction |
|  |  |  | 4.76 | -48 | -58 | 20 | Angular gyrus |
|  |  |  | 4.61 | -54 | -74 | 22 | Lateral occipital cortex, dorsa; |
|  | 2781 | R | 4.95 | 62 | 24 | 10 | Middle frontal gyrus |
|  |  |  | 4.75 | 56 | 28 | 8 | Inferior frontal gyrus |
|  |  |  | 3.95 | 36 | 26 | -24 | Frontal orbital cortex |
|  |  |  | 3.85 | 12 | 10 | 8 | Right caudate |
|  | 2247 | L | 4.20 | -40 | 22 | -18 | Temporal pole |
|  |  |  | 4.05 | -16 | -4 | -14 | Amygdala |
|  |  |  | 4.01 | -32 | 22 | -26 | Frontal orbital cortex |
|  |  |  | 3.17 | -52 | 18 | 6 | Inferior frontal gyrus |
|  | 1207 | L | 3.76 | -48 | 28 | 34 | Middle frontal gyrus |
| NCNeg > NCPos | 2451 | R | 4.04 | 0 | 52 | 32 | Superior frontal gyrus |
|  |  |  | 3.34 | -2 | 24 | 52 | Paracingulate gyrus |
|  | 1945 | R | 4.20 | 48 | -28 | -4 | Middle temporal gyrus |
|  |  |  | 3.52 | 52 | -36 | -10 | Inferior temporal gyrus |
|  | 1216 | R | 3.95 | 56 | 24 | 8 | Inferior frontal gyrus |
|  |  |  | 3.82 | 48 | 10 | 50 | Middle frontal gyrus |
|  | 859 | L | 3.71 | -46 | 24 | -2 | Inferior frontal gyrus |

**Table S2 [continuation]. Coordinates of significant activations for all conditions respect to control events (i.e. stranger in positive, negative and neutral situations) and others contrasts.** Peak activations for cooperative (Coop) and non-cooperative (NoCoop) events, for cooperative in positive situations (CPos), cooperative in negative situations (CNeg), non-cooperative in positive situations (NCPos), non-cooperative in negative situations (NCNeg). Hem.: hemisphere, R: right, L: left.

**Control tasks**

Participants performed three control-tasks in order to ensure a proper performance of the task within the scanner. Therefore, the control tasks were performed into the scanner.

- How do you feel?

Participants indicated how they felt at the beginning and the end of scanning session according to three scales: emotional valence, arousal and dominance. In emotional valence, participants reported positive valence at the beginning and the end, only two persons indicated negative valence at the end (figure S3). In emotional arousal and dominance, all participants indicated medium or high levels at the beginning and the end (figures S4 and S5).


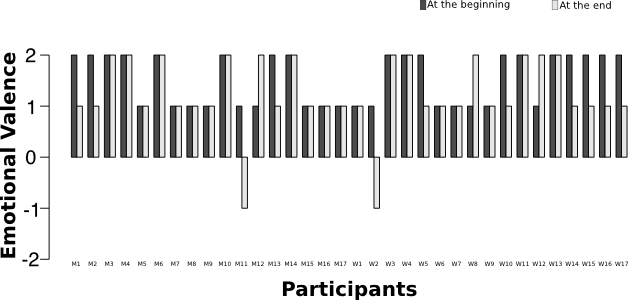


**Figure S2.** Emotional valence that participants reported at the beginning and the end of scanning session.


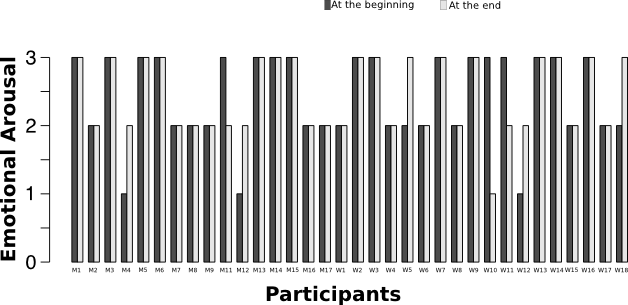


**Figure S3.** Emotional arousal that participants reported at the beginning and the end of scanning session.


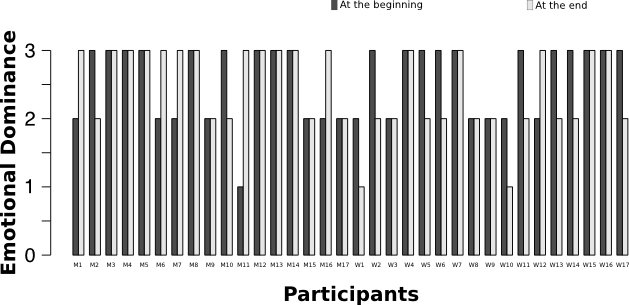


**Figure S4.** Emotional dominance that participants reported at the beginning and the end of scanning session.

- Reading comprehension and speed

Participants read a fable of Leon Tolstoi, “The falcon and the cock”, and then they answered six questions of story content. Times of reading and correct responses were registered. Participants had a reading speed (figure S6), which ensured that participants could read emotional situations (< 350), in scanning sesion in the predetermined time, and the percentage of correct responses was ≥ 75, in all participants (figure S7). Two volunteers were eliminated because their reading speed was lower than required and another one was eliminated by having a lower percentage of correct answers to 75.


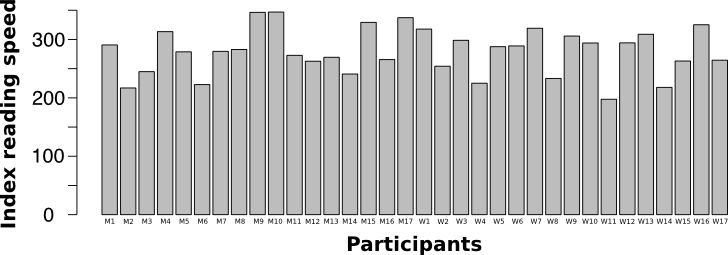


**Figure S5.** In all participants inclueded in this study, the index reading speed was inferior to 350 which ensures that could read the emotional situations in the predeterminated time for the scanning task.


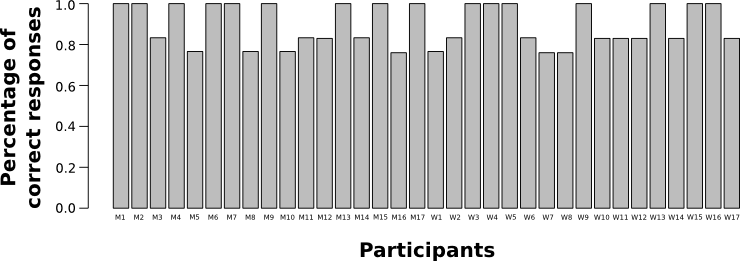


**Figure S6.** All participantes included in this study obteined a percentage of correct responses ≥ 75 which ensures that could undertand the emotional situations in the scanning task.

- Identification and face recognition

Participants viewed pictures with emotional facial expressions, i.e. sadness and happiness, and they indicated emotional valence for each pictures (six persons in two emotional expressions), responses were recorded. Then, pictures of (4) old and (4) new faces in neutral expression were presented and participants indicated if each face was old or new, responses were recorded. For identification of valence of emotional facial expression, all participants reported negative valence for sadness and positive valence for happiness (figure S8). For face recognition, percentage of correct answered was above of chance, in all participants (figure S9), just one person obtained a percentage of correct responses below of 75.


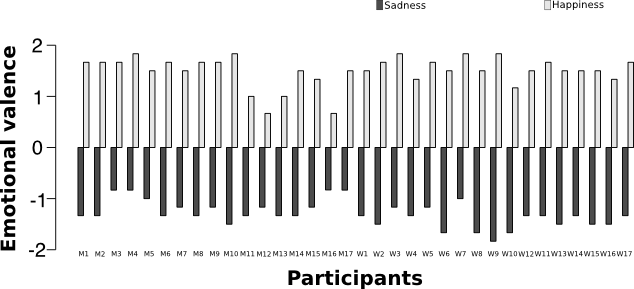


**Figure S7.** Emotional valence indicated by participants. All happy expressions obtained scores of positive valence and sad expression, scores of negative valence, which ensures that could identify emotions.


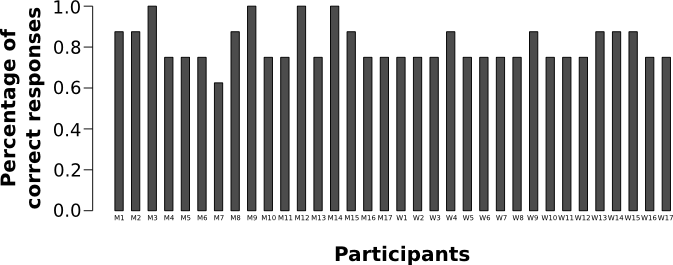


**Figure S8.** All participantes included in this study obteined a percentage of correct responses ≥ chance which ensures that could identify to confederates in the scanning task.


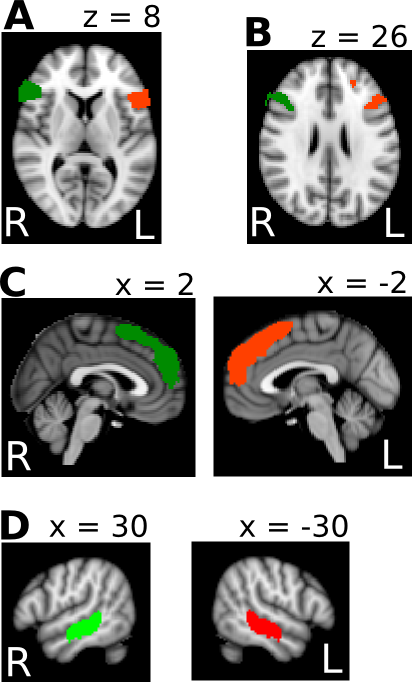


**Figure S9. ROIs.** In A**,** ROIs for IFG (inferior frontal gyrus); in B, ROIs for MFG (middle frontal gyrus); in C, ROIs for SFG (superior frontal gyrus); and in D, ROIs for MTG (middle temporal gyrus). R = right hemisphere in green, L = left hemisphere in red.

**Results**

**
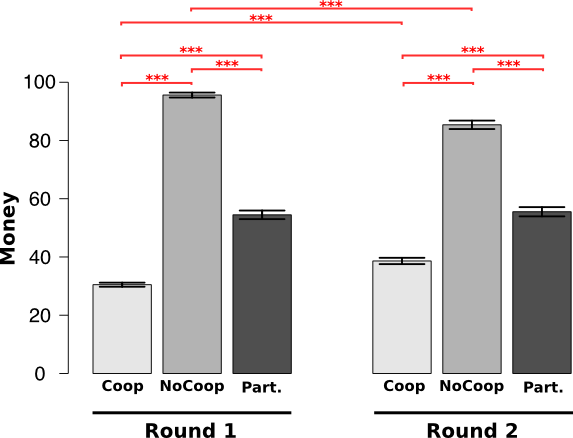
**

**Figure S10.** Earnings in each of the two rounds for three players, Coop: cooperative confederate, NoCoop: non-cooperative confederate, and Part.: participants. Bars represent the mean and the error bars show the standard error. ***p < 0.001

**Supplementary contrasts**

1. **CPos & CNeg > StrangerPos & StrangerNeg**

| **Voxels** | **P** | **Z** | **MNI coordinate** | | |  |
| --- | --- | --- | --- | --- | --- | --- |
|  |  |  | **X (mm)** | **Y (mm)** | **Z (mm)** |  |
| 4590 | 5.36e-07 | 4.48 | 8 | -94 | 2 | Right occipital pole (BA 17) |
| 3557 | 8.29e-06 | 4.85 | -10 | 56 | 28 | Left superior frontal gyrus (BA 9, 32) |
| 1855 | 0.00169 | 4.47 | -48 | -70 | 18 | Left posterior TPJ (AB 39) |
| 1408 | 0.00868 | 4.39 | -62 | -8 | -14 | Left middle temporal gyrus (BA 21, 22) |
| 1265 | 0.0152 | 3.99 | -42 | 18 | 20 | Left inferior frontal gyrus (BA 47, 8) |


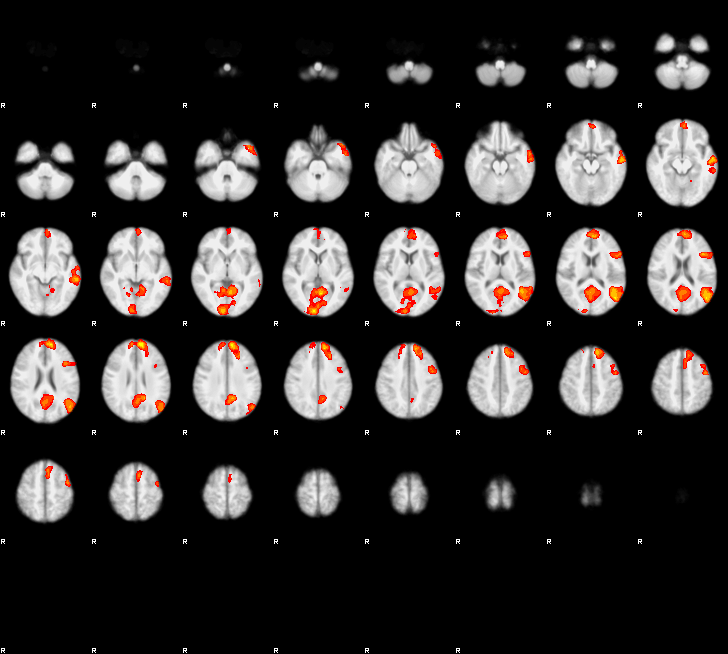


**2. NCPos & NCNeg > StrangerPos & StrangerNeg**

| **Voxels** | **P** | **Z** | **MNI coordinate** | | |  |
| --- | --- | --- | --- | --- | --- | --- |
|  |  |  | **X (mm)** | **Y (mm)** | **Z (mm)** |  |
| 3251 | 7.93e-06 | 4.91 | -6 | 56 | 14 | Superior frontal gyrus medial (BA 9) |
| 2645 | 5.63e-05 | 4.89 | 2 | -64 | 22 | Precuneous (BA 23, 26, 29, 30, 31) |
| 1699 | 0.00168 | 3.87 | -58 | -8 | -14 | Left middle temporal gyrus (BA 21, 22) |
| 1470 | 0.00416 | 4.6 | -54 | -74 | 22 | Left posterior TPJ (BA 39) |


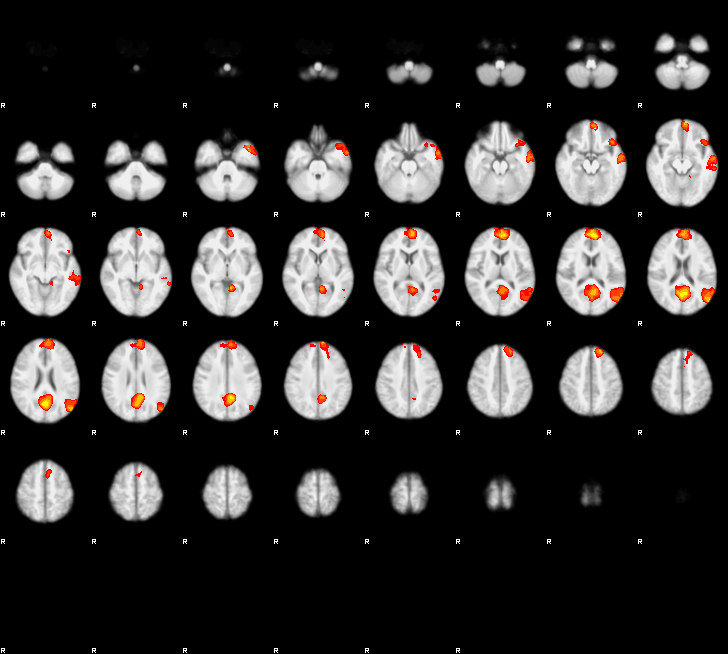


**3. CPos > StrangerPos**

| **Voxels** | **P** | **Z** | **MNI coordinate** | | |  |
| --- | --- | --- | --- | --- | --- | --- |
|  |  |  | **X (mm)** | **Y (mm)** | **Z (mm)** |  |
| 2992 | 1.44e-05 | 3.98 | 8 | -94 | 4 | Occipital cortex (BA 17) |
| 1074 | 0.0203 | 3.79 | -44 | -64 | 16 | Left posterior TPJ (BA 39) |


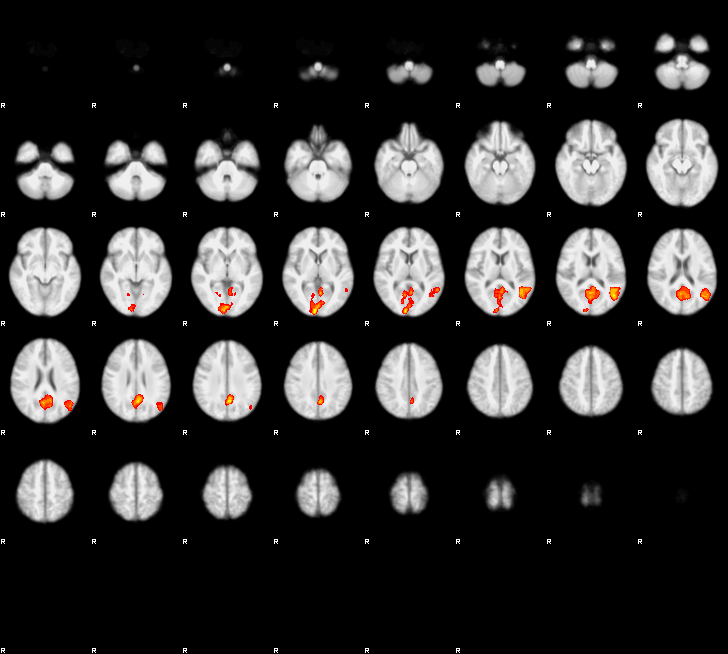


**4. CNeg > StrangerNeg**

| **Voxels** | **P** | **Z** | **MNI coordinate** | | |  |
| --- | --- | --- | --- | --- | --- | --- |
|  |  |  | **X (mm)** | **Y (mm)** | **Z (mm)** |  |
| 3166 | 2.2e-05 | 5.25 | -10 | 58 | 28 | Superior frontal gyrus medial (BA 9) |
| 3007 | 3.55e-05 | 4.25 | -50 | -68 | 20 | Left posterior TPJ (BA 39) |
| 2084 | 0.000685 | 4 | -6 | -58 | 2 | Left lingual gyrus (BA 19, 36) |


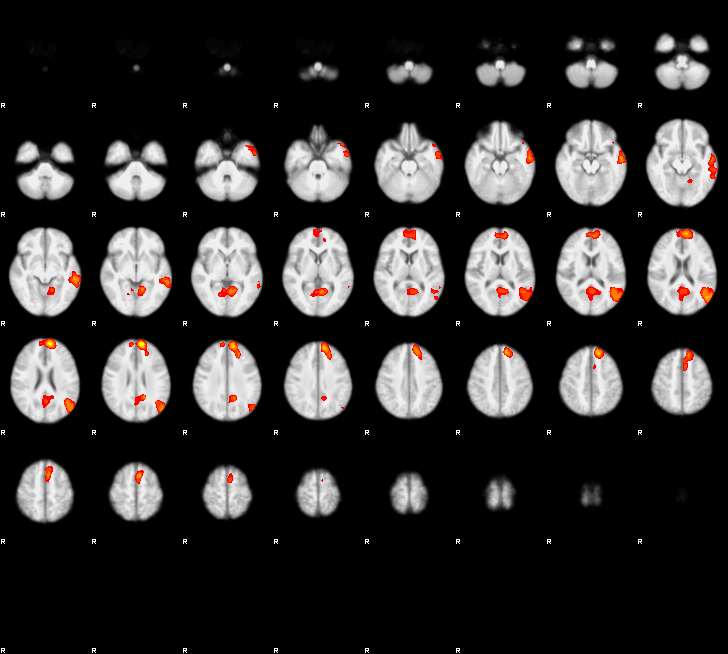


**5. NCPos > StrangerPos**

| **Voxels** | **P** | **Z** | **MNI coordinate** | | |  |
| --- | --- | --- | --- | --- | --- | --- |
|  |  |  | **X (mm)** | **Y (mm)** | **Z (mm)** |  |
| 1713 | 0.00131 | 4.13 | -18 | 44 | 44 | Superior frontal gyrus medial (BA 9) |


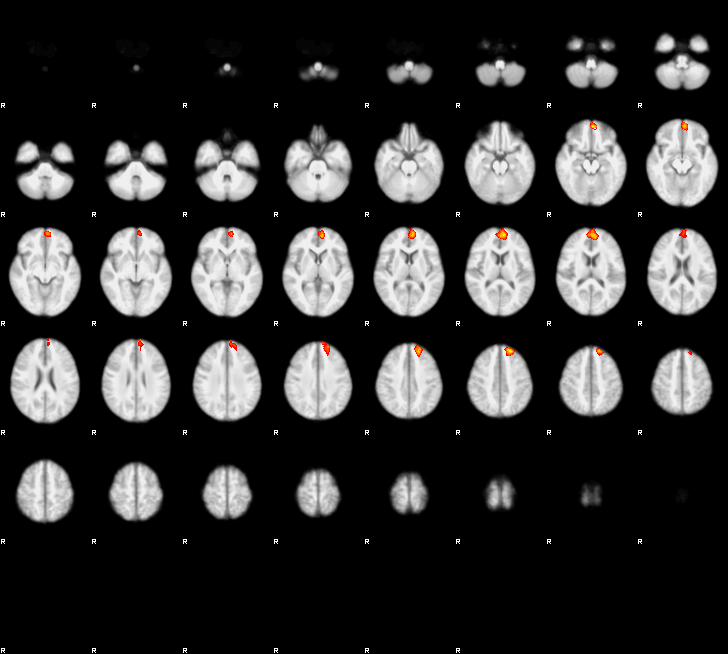


**6. NCNeg > StrangerNeg**

| **Voxels** | **P** | **Z** | **MNI coordinate** | | |  |
| --- | --- | --- | --- | --- | --- | --- |
|  |  |  | **X (mm)** | **Y (mm)** | **Z (mm)** |  |
| 3641 | 1.25e-06 | 5.1 | 0 | -64 | 24 | Precuneous (BA 23, 26, 29, 31, 31) |
| 2257 | 0.000137 | 4.44 | -6 | 60 | 20 | Superior frontal gyrus medial (BA 9) |
| 1587 | 0.00185 | 4.56 | -48 | -70 | 22 | Left posterior TPJ (BA 39) |
| 1103 | 0.0154 | 4.01 | -58 | -4 | -16 | Left middle temporal gyrus (BA 21, 22) |


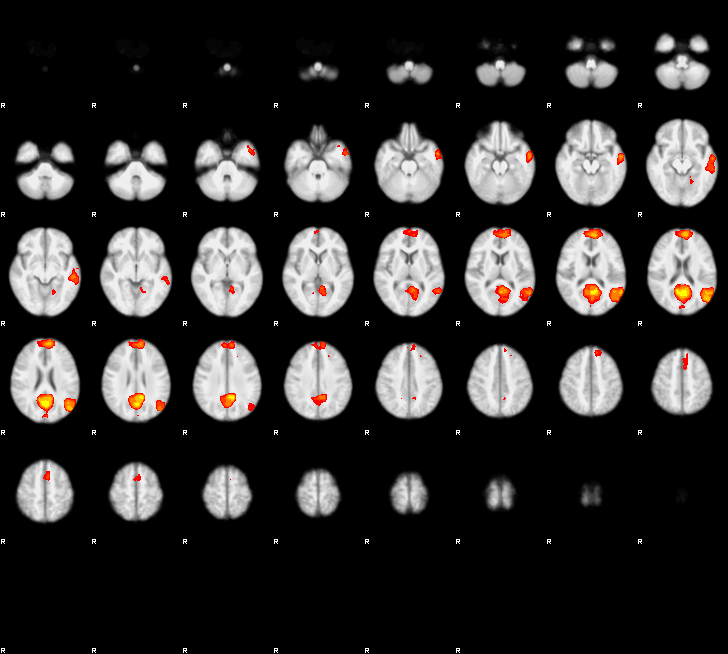


**7. CPos & CNeg & NCPos & NCNeg > StrangerPos & StrangerNeg**

| **Voxels** | **P** | **Z** | **MNI coordinate** | | |  |
| --- | --- | --- | --- | --- | --- | --- |
|  |  |  | **X (mm)** | **Y (mm)** | **Z (mm)** |  |
| 4816 | 4.17e-07 | 5.44 | -4 | 54 | 16 | Superior frontal gyrus, medail (BA 9, 32) |
| 3872 | 4.59e-06 | 4.84 | -10 | -50 | 32 | Precuneous (BA 23, 31) |
| 2877 | 7.63e-05 | 4.54 | -64 | -32 | -8 | Left middle temporal gyrus (BA 21, 22) |
| 2070 | 0.000959 | 4.74 | -48 | -70 | 20 | Left posterior TPJ (BA 39) |


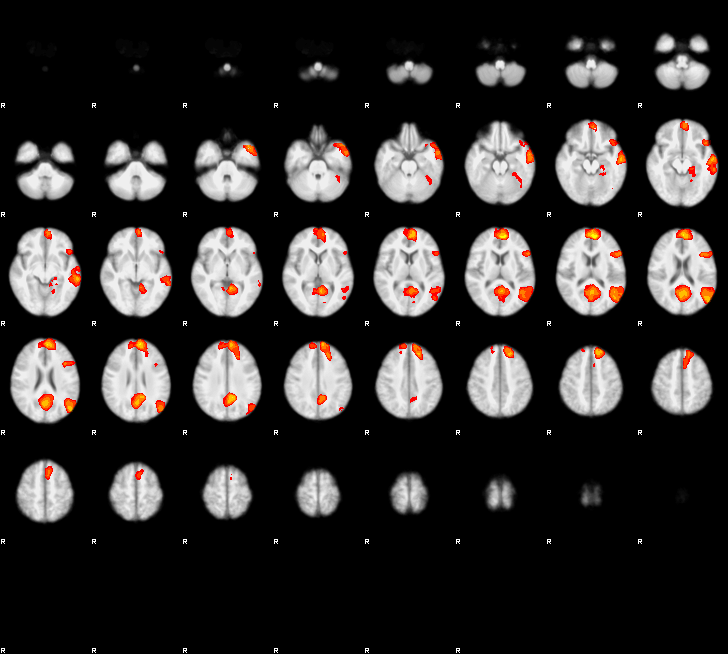


**8. CPos & NCPos > StrangerPos**

| **Voxels** | **P** | **Z** | **MNI coordinate** | | |  |
| --- | --- | --- | --- | --- | --- | --- |
|  |  |  | **X (mm)** | **Y (mm)** | **Z (mm)** |  |
| 2136 | 0.000378 | 4.61 | -2 | 56 | 14 | Superior frontal gyrus, medail (BA 9, 32) |
| 1870 | 0.000991 | 4.33 | -4 | -58 | 32 | Precuneous (BA 23, 26, 29, 31, 31) |
| 974 | 0.038 | 3.53 | -50 | -68 | 20 | Left posterior TPJ (BA 39) |


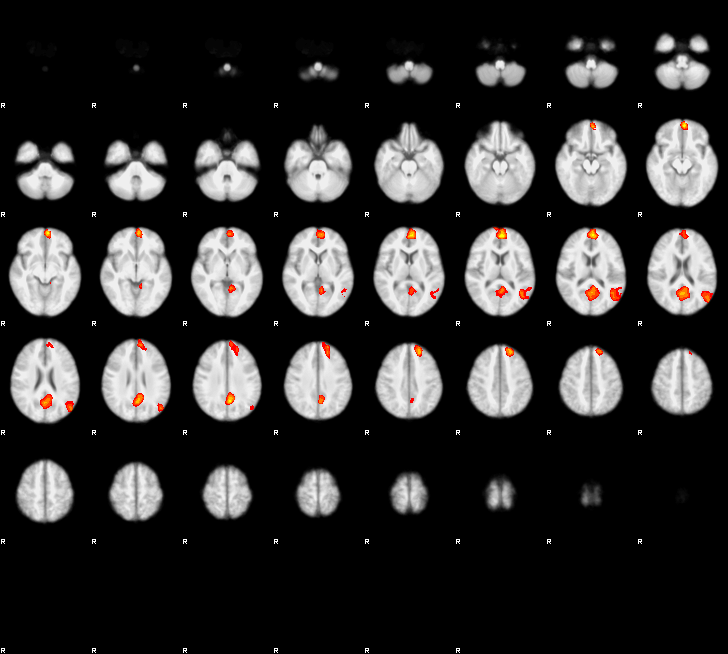


**9. CNeg & NCNeg > StrangerNeg**

| **Voxels** | **P** | **Z** | **MNI coordinate** | | |  |
| --- | --- | --- | --- | --- | --- | --- |
|  |  |  | **X (mm)** | **Y (mm)** | **Z (mm)** |  |
| 4708 | 2.38e-07 | 5.17 | -48 | -72 | 22 | Left posterior TPJ (BA 39) |
| 3922 | 2.15e-06 | 5.45 | -10 | 56 | 28 | Left superior frontal gyrus medial (BA 9) |
| 3866 | 2.56e-06 | 4.8 | -12 | -52 | 32 | Precuneous (BA 23, 31) |


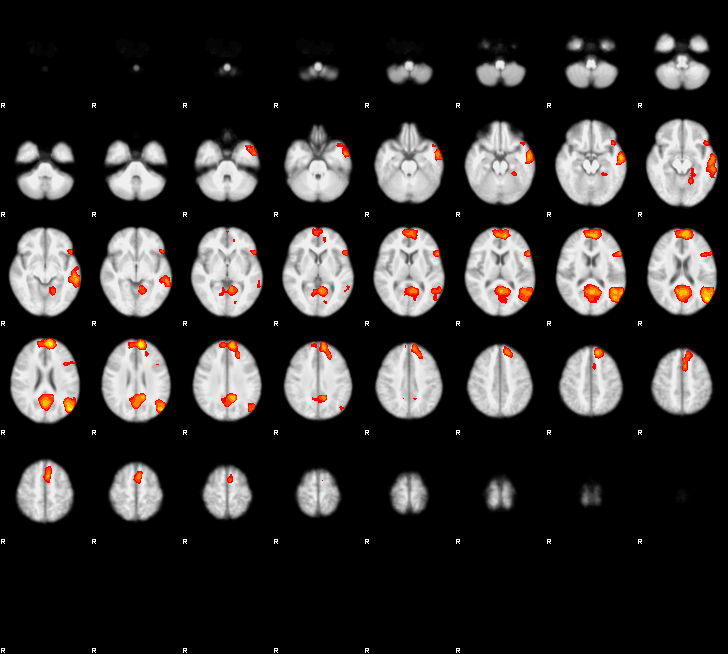


**10. StrangerPos > StrangerNeg**

| **Voxels** | **P** | **Z** | **MNI coordinate** | | |  |
| --- | --- | --- | --- | --- | --- | --- |
|  |  |  | **X (mm)** | **Y (mm)** | **Z (mm)** |  |
| 1032 | 0.0228 | 3.43 | 24 | 64 | 2 | Right frontal pole (BA 10) |


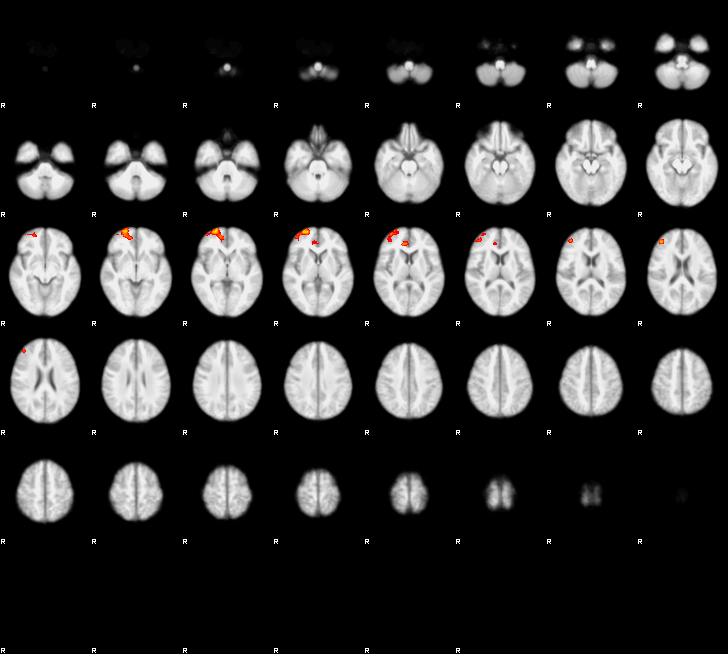


**11. StrangerNeg > StrangerPos**

| **Voxels** | **P** | **Z** | **MNI coordinate** | | |  |
| --- | --- | --- | --- | --- | --- | --- |
|  |  |  | **X (mm)** | **Y (mm)** | **Z (mm)** |  |
| 1279 | 0.00745 | 4.02 | 4 | 60 | 20 | Right frontal pole, medial (BA 10) |


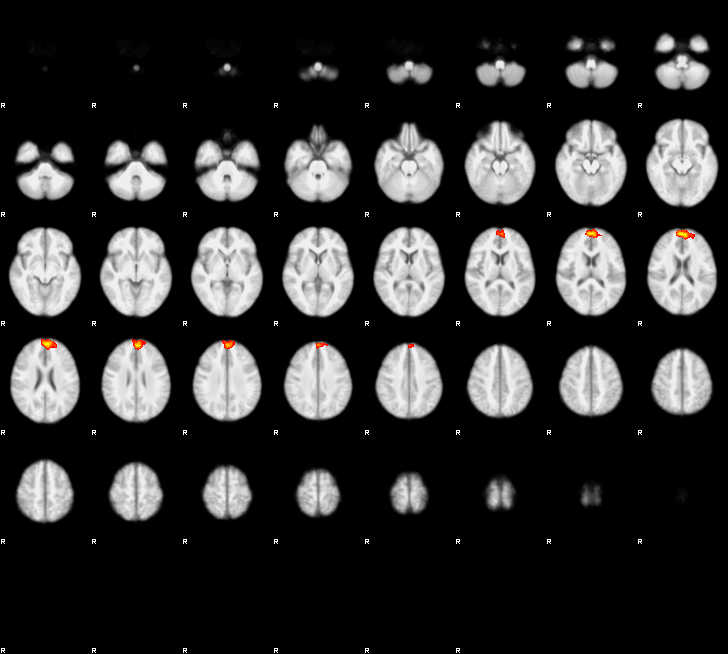


**12. StrangerPos > StrangerNeutral**

| **Voxels** | **P** | **Z** | **MNI coordinate** | | |  |
| --- | --- | --- | --- | --- | --- | --- |
|  |  |  | **X (mm)** | **Y (mm)** | **Z (mm)** |  |
| 8219 | 6.7e-12 | 4.75 | 38 | 54 | -4 | Orbital frontal cortex (BA 10, 11) |
| 4226 | 2.38e-07 | 5.03 | 0 | -32 | 24 | Precuneous (BA 23, 26, 29, 31, 31) |
| 2669 | 3.43e-05 | 4.87 | -42 | -62 | 48 | Left dorsal TPJ (7, 39) |
| 1966 | 0.000439 | 4.56 | 48 | -50 | 38 | Right dorsal TPJ (7, 39) |


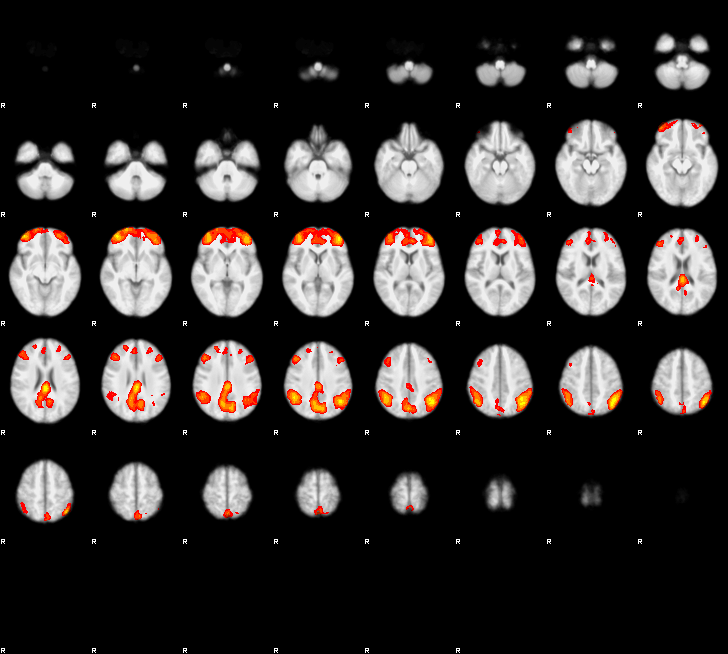


**13. StrangerNeg > StrangerNeutral**

| **Voxels** | **P** | **Z** | **MNI coordinate** | | |  |
| --- | --- | --- | --- | --- | --- | --- |
|  |  |  | **X (mm)** | **Y (mm)** | **Z (mm)** |  |
| 6748 | 9.17e-11 | 5.48 | 4 | 62 | 14 | Frontal pole (BA 10) |
| 2667 | 2.08e-05 | 4.7 | 2 | -58 | 32 | Precuneous (BA 23, 31) |
| 2606 | 2.59e-05 | 3.81 | 50 | 30 | 0 | Right inferior frontal gyrus (BA 45, 47) |
| 2447 | 4.64e-05 | 4.91 | -44 | -58 | 52 | Left dorsal TPJ (7, 39) |
| 2249 | 9.76e-05 | 4.9 | -12 | 4 | 10 | Left caudate |
| 1114 | 0.0118 | 3.81 | 54 | -52 | 26 | Right TPJ (BA 39) |
| 940 | 0.0278 | 4.42 | 64 | -26 | -10 | Right middle temporal gyrus (BA 21, 22) |
| 829 | 0.0489 | 4.48 | -44 | 20 | 38 | Left middle temporal gyrus (BA 21, 22) |


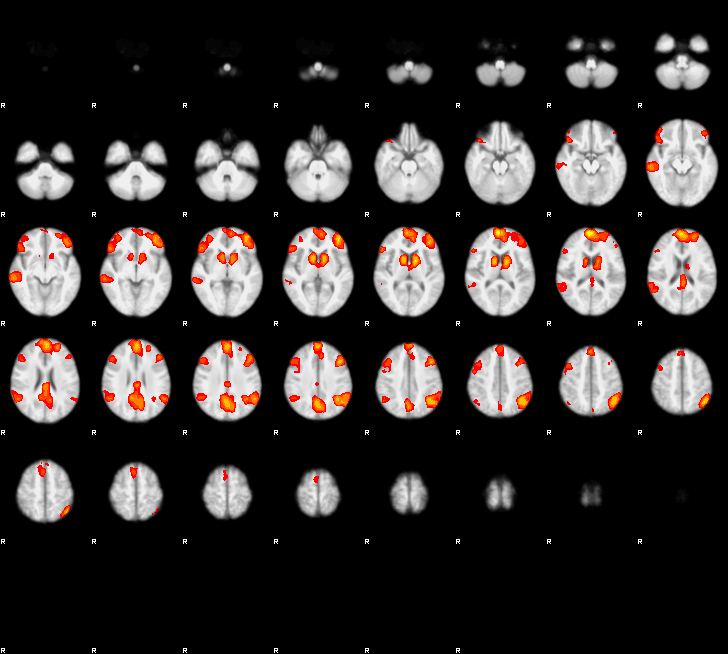


**14. StrangerPos & StrangerNeg > StrangerNeutral**

| **Voxels** | **P** | **Z** | **MNI coordinate** | | |  |
| --- | --- | --- | --- | --- | --- | --- |
|  |  |  | **X (mm)** | **Y (mm)** | **Z (mm)** |  |
| 9833 | 6.49e-14 | 5.34 | -42 | 48 | 4 | Left frontal pole (BA 11, 47) |
| 4143 | 1.79e-07 | 5.1 | 0 | -30 | 24 | Precuneous (BA 23, 31) |
| 2879 | 1.14e-05 | 5.35 | -42 | -60 | 54 | Left posterior TPJ (BA 7, 39) |
| 1910 | 0.000411 | 4.23 | 10 | 14 | 4 | Right caudate |
| 1790 | 0.000666 | 4.33 | 48 | -50 | 36 | Right TPJ (BA 39) |
| 1094 | 0.014 | 3.92 | 44 | 26 | 36 | Right middle frontal gyrus (BA 46) |


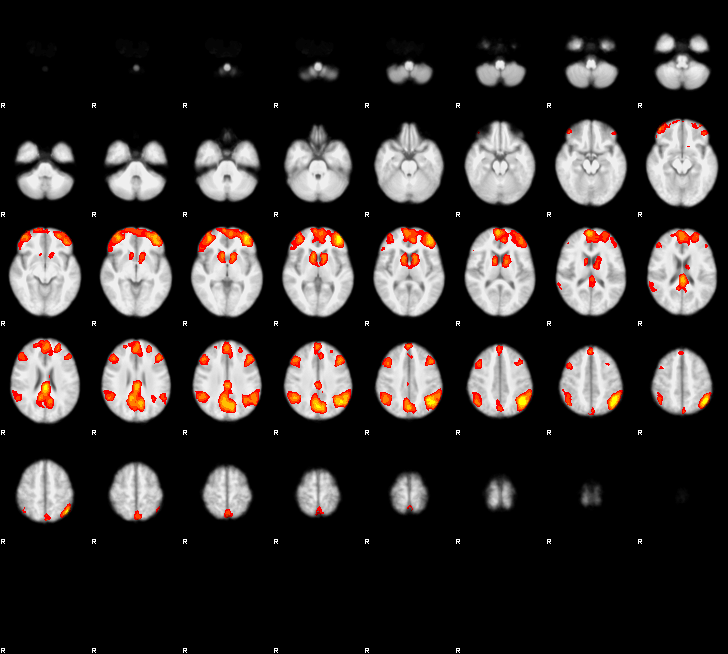

Supplement: Supplementary file 1 [file Table_1.docx]
